# Genomic structure of a crossbred Landrace pig population

**DOI:** 10.1371/journal.pone.0212266

**Published:** 2019-02-28

**Authors:** Letícia Borges Joaquim, Tatiane Cristina Seleguim Chud, Jorge Augusto Petroli Marchesi, Rodrigo Pelicioni Savegnago, Marcos Eli Buzanskas, Ricardo Zanella, Mauricio Egidio Cantão, Jane Oliveira Peixoto, Mônica Correa Ledur, Renato Irgang, Danísio Prado Munari

**Affiliations:** 1 Universidade Estadual Paulista (Unesp), Faculdade de Ciências Agrárias e Veterinárias, Departamento de Ciências Exatas, Jaboticabal, São Paulo, Brazil; 2 Universidade Federal da Paraíba (UFPB), Departamento de Zootecnia, Areia, Paraíba, Brazil; 3 Universidade de Passo Fundo (UPF), Passo Fundo, Rio Grande do Sul, Brazil; 4 Embrapa Suínos e Aves, Concórdia, Santa Catarina, Brazil; 5 Universidade Federal de Santa Catarina (UFSC), Departamento de Zootecnia e Desenvolvimento Rural, Centro de Ciências Agrárias, Florianópolis, Santa Catarina, Brazil; National Cheng Kung University, TAIWAN

## Abstract

Single nucleotide polymorphism (SNP) markers are used to study population structure and conservation genetics, which permits assessing similarities regarding the linkage disequilibrium and information about the relationship among individuals. To investigate the population genomic structure of 300 females and 25 males from a commercial maternal pig line we analyzed linkage disequilibrium extent, inbreeding coefficients using genomic and conventional pedigree data, and population stratification. The average linkage disequilibrium (r^2^) was 0.291 ± 0.312 for all adjacent SNPs, distancing less than 100 Kb (kilobase) between markers. The average inbreeding coefficients obtained from runs of homozygosity (ROH) and pedigree analyses were 0.119 and 0.0001, respectively. Low correlation was observed between the inbreeding coefficients possibly as a result of genetic recombination effect accounted for the ROH estimates or caused by pedigree identification errors. A large number of long ROHs might indicate recent inbreeding events in the studied population. A total of 36 homozygous segments were found in more than 30% of the population and these ROH harbor genes associated with reproductive traits. The population stratification analysis indicated that this population was possibly originated from two distinct populations, which is a result from crossings between the eastern and western breeds used in the formation of the line. Our findings provide support to understand the genetic structure of swine populations and may assist breeding companies to avoid a high level of inbreeding coefficients to maintain genetic diversity, showing the effectiveness of using genome-wide SNP information for quantifying inbreeding when the pedigree was incomplete or incorrect.

## Introduction

The availability of low-cost single nucleotide polymorphism (SNP) panels has become an efficient tool for evaluation of the genetic structure in livestock populations, such as cattle, poultry, and pigs. The knowledge of the linkage disequilibrium (LD) pattern can reveal the population history, as well as characterize the genetic diversity [1].

Controlling inbreeding has fundamental importance in animal production and conservation genetics because it leads to higher homozygosity, which leads to reduced genetic diversity and fitness [2]. Loss of genetic diversity has been reported in livestock breeds, for example, Vicente et al. [3] described the level of inbreeding as the major concern in native Portuguese swine breeds. Therefore, studying the loss of genetic diversity is necessary for sustainable improvement in swine populations [4]. Traditionally, the relationship among individuals are calculated from pedigree data and collateral relationships do not increase inbreeding, however, there are some disadvantages in this method, such as the inability for capturing the effect of distant relatives and the disregard of the meiotic process [5].

Inbreeding coefficients also can be estimated using genotype data, based on genomic-pedigree information [6] or calculated by the levels of homozygosity [5]. Runs of homozygosity (ROH) are contiguous homozygous stretches in an offspring genome [2]. The ROH is an alternative to estimate populations inbreeding coefficients due to more accurate detection of recessive and rare mutations compared to other methods. Furthermore, ROH detection permits describing the demographic history and domestication events in a population.

Previous studies have used SNP chip data to evaluate genetic diversity and conservation of commercial and local pigs [7–9]. Studies of the genetic structure using SNP genotyping data can provide information about the genetic ancestry of individuals and on the evolutionary history of pig’s populations that may assist breeders on mating schemes to maintain the genetic variation in swine lines. The objectives of this study were to investigate the genomic structure and population stratification in a maternal line of swine by means of the linkage disequilibrium, inbreeding coefficients, ROHs, and to determine the population stratification.

## Materials and methods

### Animals and DNA extraction

Tissue samples were collected from ear puncturing of the 300 females and 25 males of a maternal line of pigs. The animals were obtained from a swine breeding program located in Santa Catarina, Brazil. This line was initially developed from the crosses of eastern and western breeds and the expected proportion of the genes were 50% Landrace, 12.50% Meishan, 12.50% Xia Jing, 8.33% Hampshire, 8.33% Pietrain and 8.33% Large White. Genomic DNA was extracted from 200 mg of the harvested tissue, with PureLink Genomic DNA Mini Kit (Invitrogen, Calrsbad, USA) following the manufacturer protocol. DNA quantity, quality, and integrity were assessed using the NanoDrop 1000 Spectrophotometer (Thermo Scientific, Waltham, MA, USA) and the samples read with 260/280 nm between 1.75–1.95 were diluted to a final concentration of 50 ng/μl.

#### Genotyping and quality control of samples and SNPs

The animals were genotyped using the Illumina PorcineSNP60-v2 BeadChip, comprising 61,565 SNPs distributed throughout the porcine genome. Genotype quality control was performed using PLINK v.1.9 [10] to remove samples and SNPs with call rate lower than 90%. According to Bosse et al. [2] no additional filters, such as minor allele frequency were applied in this study.

#### Linkage disequilibrium

The LD was measured using the r^2^ equation:

$$r^{2}=\frac{D^{2}}{f(A)f(a)f(B)f(b)}$$

where f(A), f(a), f(B), and f(b) are the respective frequencies of alleles A, a, B and b, and D is f(AB)−f(A)f(B), as proposed by Hill and Robertson [11]. For all pairs of autosomal SNPs, the r^2^ measures were calculated using the parameters—ld-window 60,000—ld-window-r2 0 of PLINK [10]. The r^2^ was calculated for all adjacent SNPs in 60,000 Kb windows.

### Inbreeding coefficient based on pedigree and genomic data

The inbreeding coefficient using pedigree data (F_PED_) on three generations of a swine maternal line population was estimated following the methodology proposed by Wright (1931) [12]. Inbreeding estimates were also calculated using runs of homozygosity (F_ROH_) for autosomal SNPs by means of the PLINK v.1. [10]. SNPs located on sex chromosomes were excluded because recombination on these chromosomes is different from the autosomes, and also because the genetic map resolution for the X-chromosome differed from the autosomes in the pig genome [2]. The ROHs were defined as segments with at least 50 homozygous SNPs at a minimum distance of 1,000 Kb per animal, allowing one heterozygous SNP and one missing SNP within a 50 SNPs window [9].

From the identification of ROHs, the inbreeding coefficient for each genomic individual (F_ROH_) was estimated using the following equation:

$$F_{ROH}=\frac{\sum L_{ROH}}{L}$$

in which ∑*L_ROH_* is the sum of all ROHs identified for each individual by length (*L*) of the pig genome (2,808,525 Kb, Scrofa10.2, may 2014). The ROH frequencies were calculated for all ROHs identified in the population and then compared to verify how many individuals shared the same ROHs (same start and end of the ROH positions).

The ROH regions observed in more than 30% of the samples were investigated to identify possible conserved regions and determine the genes that might be associated with adaptive traits such as survival and disease resistance. Genes located on ROH regions were identified using the UCSC genome browser (Scrofa 10.2) and Ensembl ID gene was used for the enrichment analysis. Gene ontology (GO) enrichment analysis was performed using David 6.8 database (https://david.ncifcrf.gov/home.jsp) and the results were considered statistically significant at FDR cutoff < 0.20. For visualization and reduction of redundant GO-terms, the REVIGO software [13] was used.

### Population structure

Population stratification analyses considering the admixture model were carried out using STRUCTURE software v.2.3.4 [14]. The K values (number of populations or genetic groups) varying from one to eight were estimated using a Bayesian approach considering 10,000 iterations and a burn-in period of 1,000 cycles. The analyses were repeated ten times for each K while the correct number of K was estimated using the delta-k statistics, which is based on the shift rate in the likelihood logarithm between successive values of K [15]. Delta-k was calculated using the STRUCTURE HARVESTER software v.0.6.94 [16].

In addition, the level of ancestry divergence among populations (Fst) was obtained using the ADMIXTURE software [17], which the likelihood method was applied to estimate the ancestry matrix coefficients. Principal component analysis was performed for the population using PLINK v.1.9 [10]. Analyses were carried out with all SNPs and also excluding SNPs with r^2^ greater than 0.20 to eliminate redundancy between markers in high LD.

## Results

After the genotype quality control, one animal and 3,795 SNPs were excluded due of the low call rate. Therefore, 57,770 SNPs were analyzed, including 1,498 SNPs on chromosome X (SSCX), 15 SNPs on chromosome Y (SSCY), and 4,076 SNPs with no defined position (Table 1).

**Table 1:** Descriptive analysis of SNP markers along the genome per chromosome.

**CHR**	**Size** **CHR**^2^	**N SNPs/CHR**^3^	**%** **SNP/CHR**^4^	**N SNPs mono.**^5^	**% SNP** **mono.**^6^	**MAF**^7^
0^1^	-	4076	7,06	365	8,95	0,2527
SSC1	315,32	6624	11,47	748	11,29	0,2313
SSC2	162,57	3321	5,75	259	7,80	0,2510
SSC3	144,79	2841	4,92	243	8,55	0,2454
SSC4	143,47	3541	6,13	456	12,88	0,2385
SSC5	111,51	2360	4,09	266	11,27	0,2386
SSC6	157,77	3215	5,57	256	7,96	0,2594
SSC7	134,76	3297	5,71	314	9,52	0,2366
SSC8	148,49	2795	4,84	253	9,05	0,2513
SSC9	153,67	3228	5,59	227	7,03	0,2529
SSC10	79,10	1752	3,03	128	7,31	0,2661
SSC11	87,69	1862	3,22	151	8,11	0,2476
SSC12	63,59	1560	2,70	104	6,67	0,2545
SSC13	218,64	4072	7,05	367	9,01	0,2604
SSC14	153,85	3860	6,68	329	8,52	0,2701
SSC15	157,68	2950	5,11	334	11,32	0,2328
SSC16	86,90	1869	3,24	159	8,51	0,2566
SSC17	69,70	1693	2,93	138	8,15	0,2242
SSC18	61,22	1341	2,32	103	7,68	0,2455
SSCX	144,29	1498	2,59	297	19,83	0,1898
SSCY	1,64	15	0,03	0	0	0

^1^ Chromosome 0 represents non-defined position

^2^ Chromosome size in the Megabase

^3^ Number of SNPs per chromosome

^4^ SNP percentage per chromosome

^5^ Number of monomorphic SNPs

^6^ Percentage of monomorphic SNPs

^7^ The average for the minor allelic frequency per chromosome.

### Linkage disequilibrium

**Fig 1:**
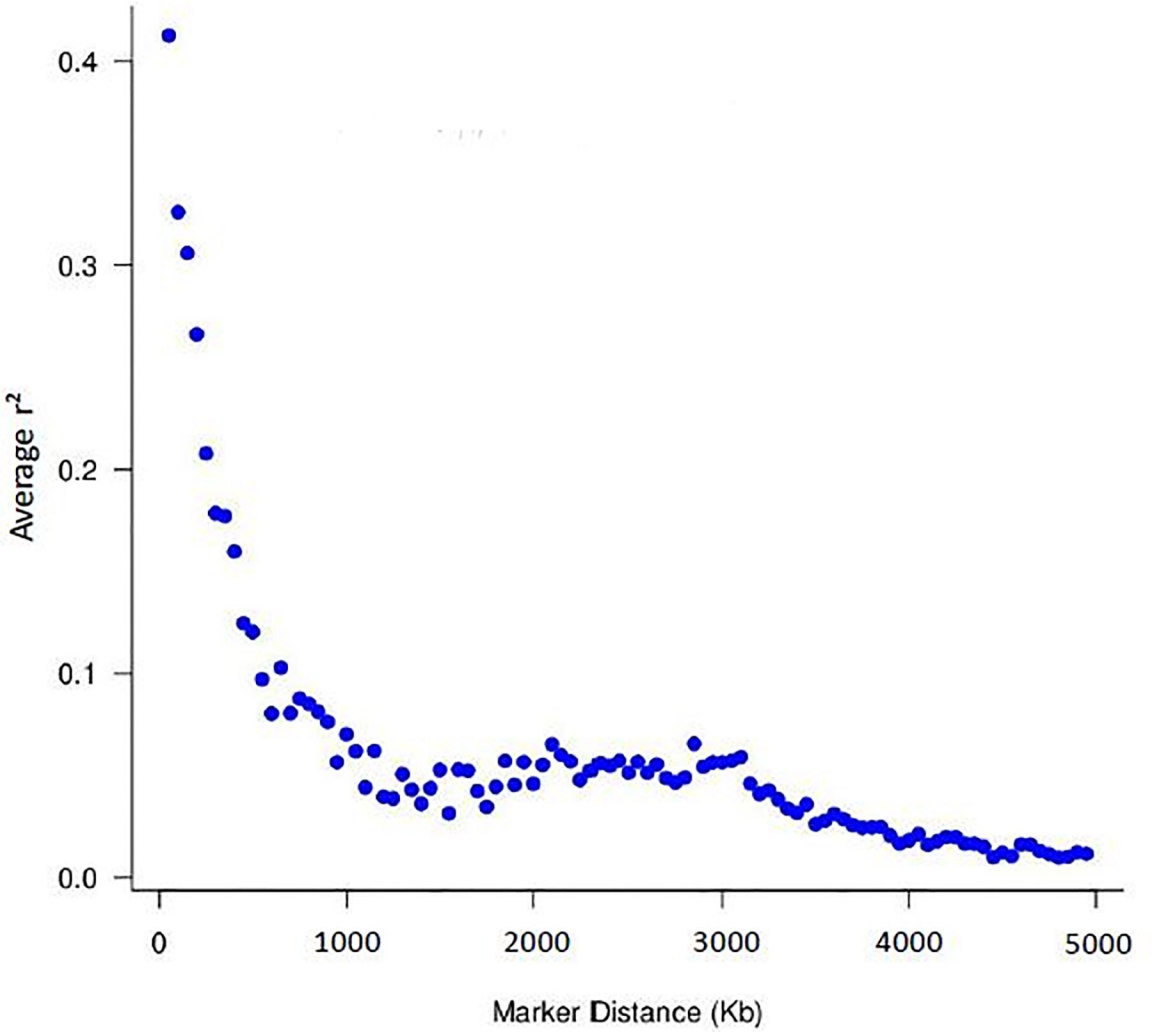
Decay of average linkage disequilibrium (r^2^) over distance (Kb) between markers for a commercial maternal line.

A total of 52,196 SNPs were used to calculate the average LD (r^2^) between all adjacent SNPs, with distance less than 100 Kb. The average and standard deviation of estimated r^2^ was 0.29 ± 0.31, indicating high variability around the mean value estimated for the LD. It was observed that as the distance between markers increased, the LD values decreased, and for distances greater than 1 Mb, the mean r^2^ was less than 0.20 (Fig 1). The mean r^2^ converged to 0.30, considered a strong LD and useful for QTL mapping, approximately at 200 to 300 Kb.

### Inbreeding coefficient

**Fig 2:**
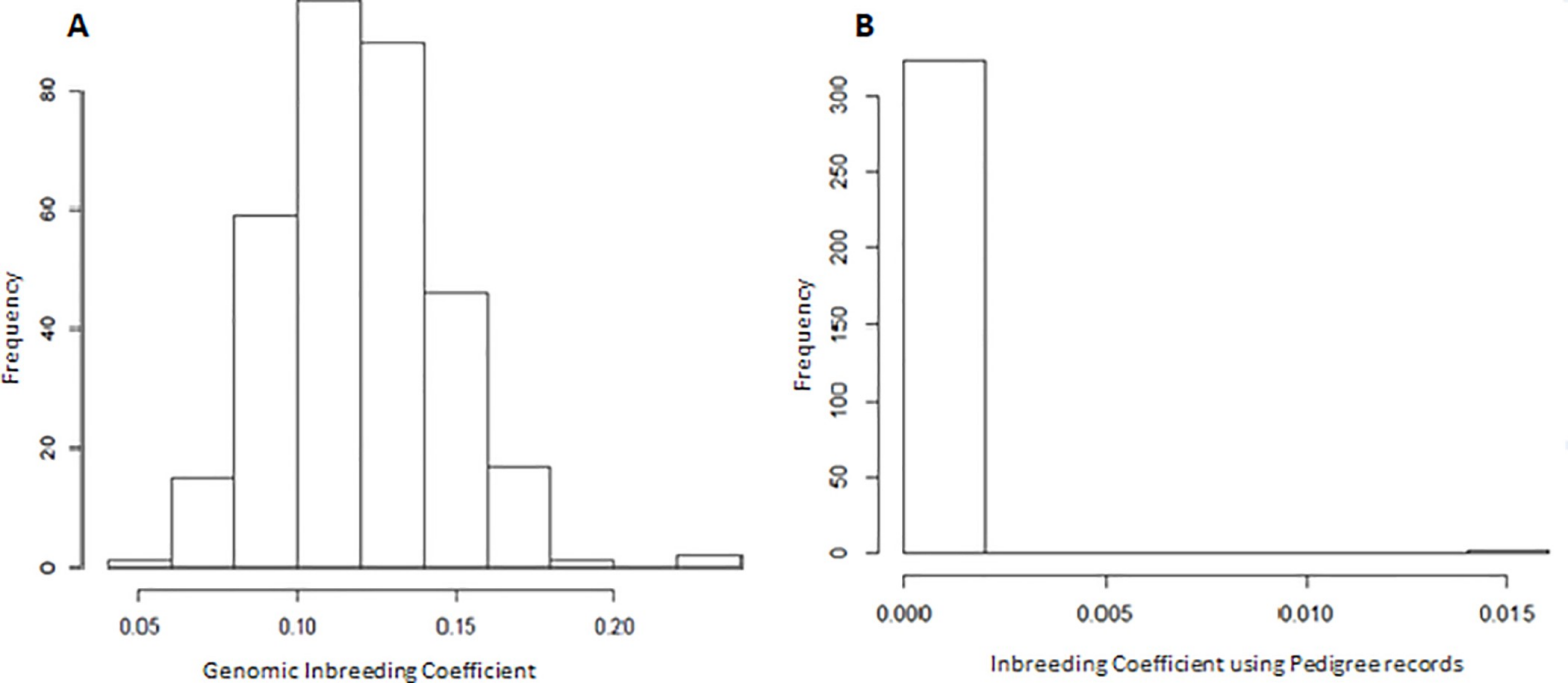
Inbreeding coefficient of the commercial maternal line using genomic data (A) and pedigree records (B).

ROH covered 11.89% of the genome of this population with an average of 54 ROH per animal and a maximum number of 74 ROH. The size ranged between 152,110 Kb and 652,500 Kb, with an average of 333,900 Kb. The value of F_ROH_ was 0.119, with 0.054 and 0.232 as minimum and maximum values, respectively. The pedigree analysis resulted in an average F_PED_ of 0.00011, ranging from 0 to 0.015 and with a low correlation (0.04) between F_ROH_ and F_PED_ (Fig 2).

### Shared ROH

**Fig 3:**
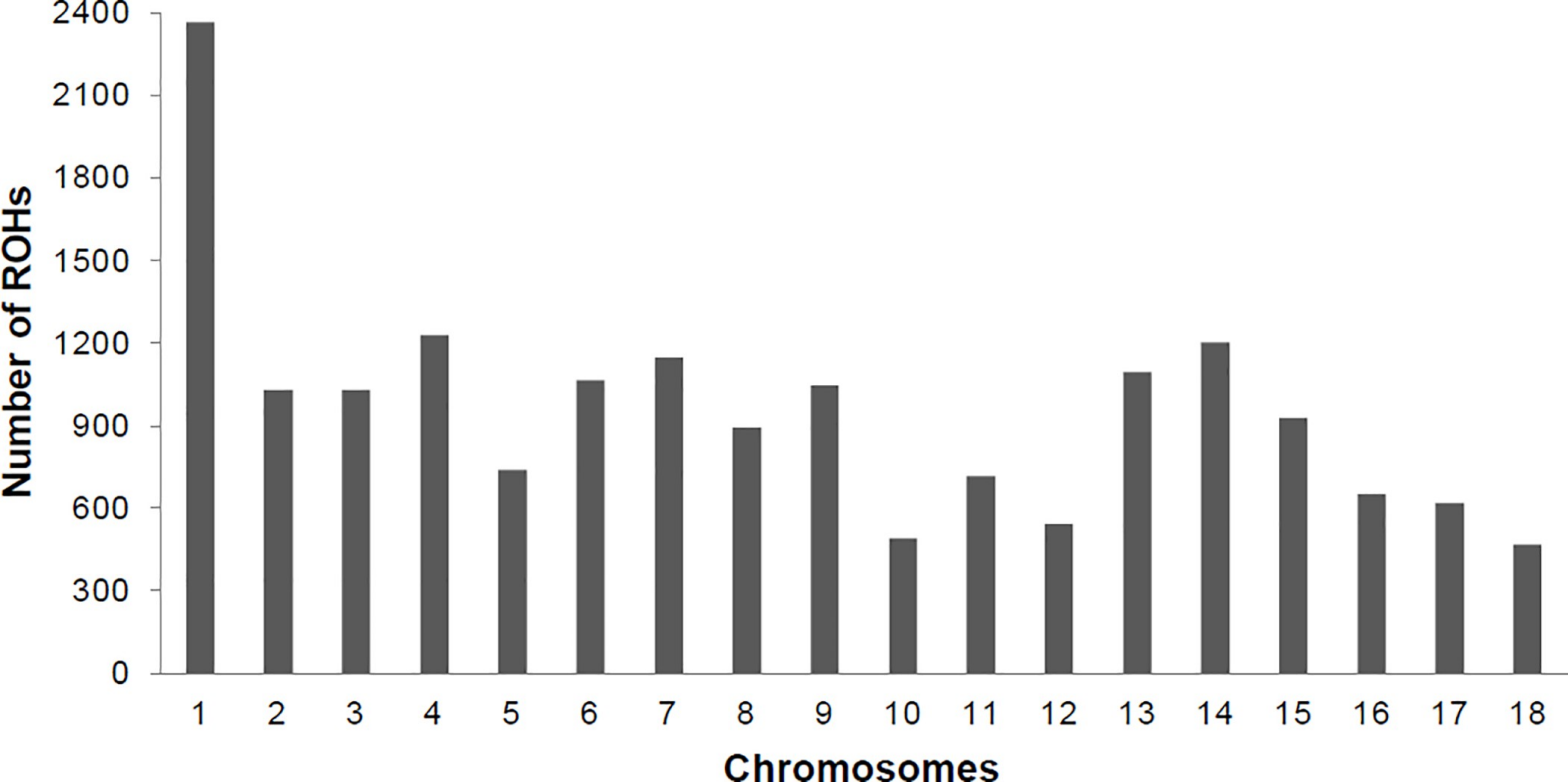
Number of runs of homozygosity (ROH) shared between individuals per chromosome of commercial maternal line.

**Fig 4:**
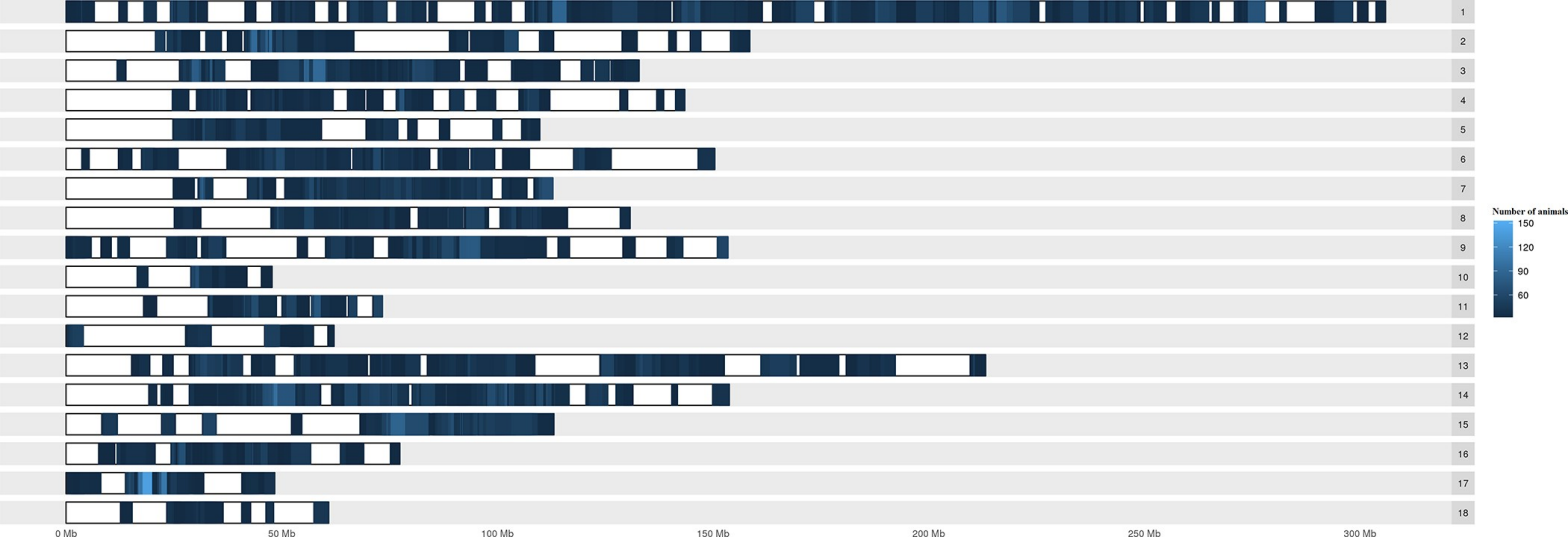
Karyogram of runs of homozygosity (ROH) shared among individuals considering classes according number of animals.

No identical segments were observed to be completely shared among all the individuals. The chromosomes SSC1, SSC4, SSC7, and SSC14 were those with the largest number of shared ROHs in this population (Figs 3 and 4). The SSC1 stood out with more than 1,000 shared ROH among chromosomes SSC1, SSC4, SSC7, and SSC14.

A total 36 homozygous segments were observed in more than 30% of the population. The ROHs identified had a small size (1 to 5 Mb) and most of these segments (78%) were located on SSC17. Other ROHs observed in more than 30% of the population were on SSC1, SSC4, SSC7 and SSC14.

**Fig 5:**
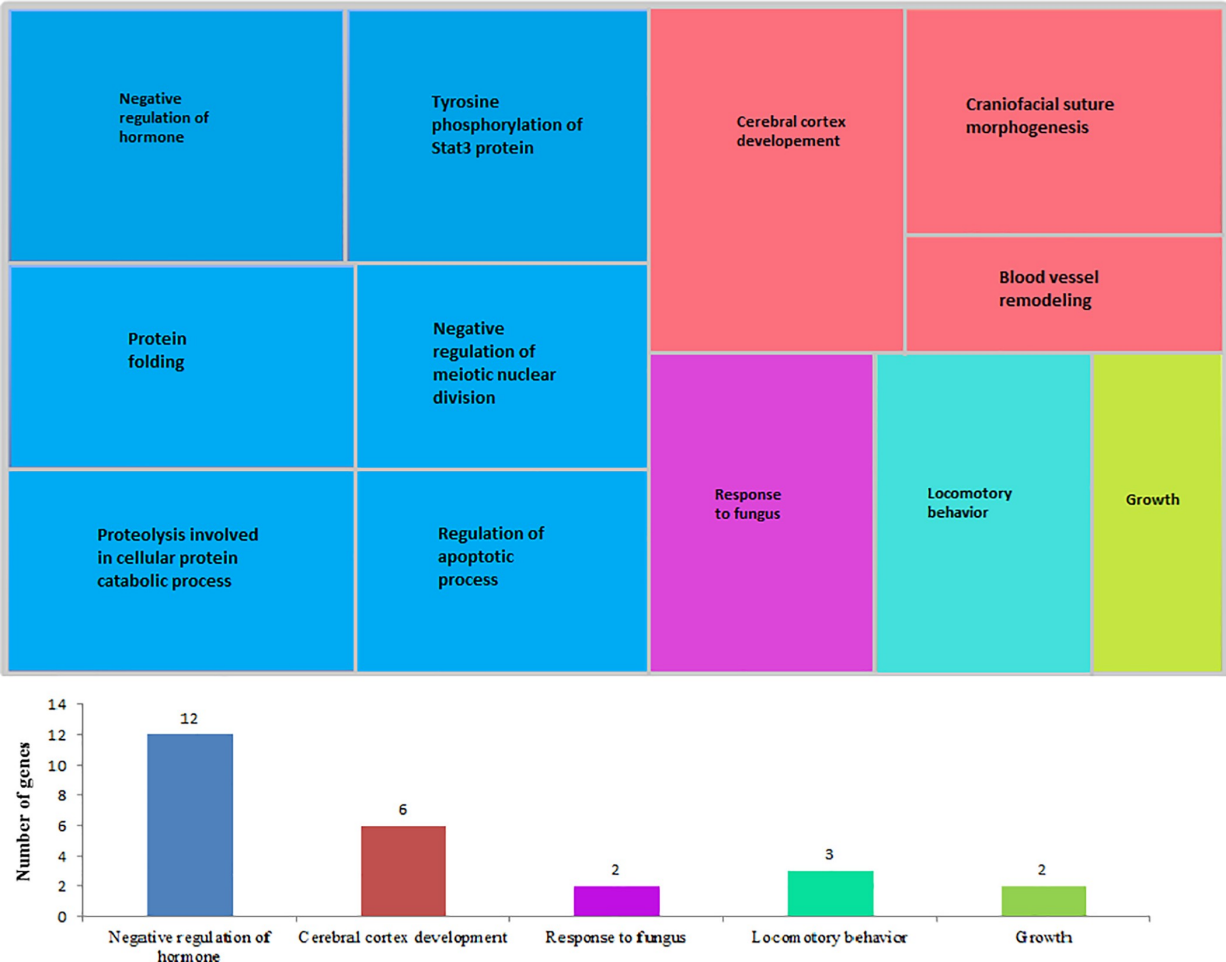
“Treemap” of biological processes based on the Gene Ontology terms statistically significant (FDR < 0.20) for genes identified in ROHs shared in the population. Each rectangle is a single cluster representative. The representatives are joined into ‘superclusters’ of related terms, visualized with different colors.

A total of 240 genes were found in the homozygous regions with a frequency greater than 30%. Before the GO enrichment analysis, 26 genes were identified in 17 enriched GO-terms associated with biological processes (FDR < 0.20; S1 Table), which were then reduced by redundancy to 12 GO-terms grouped in five superclusters (Fig 5). The most enriched supercluster harboring the largest number of genes was the negative regulation of hormone secretion, followed by cerebral cortex development. These biological processes may be related to the domestication process of the studied animals. The response to fungus (GO:0009620), enriched with the genes *IL25* (interleukin 25) and *MALT1* (MALT1 paracaspase) (S1 Table; Fig 5), may be involved with survival traits such as resistance to diseases.

The region between 48,283,163 and 50,313,732 bp (base pair) on SSC14 was shared among 97 individuals (30%) and harbor the gene *LIF* (*Leukemia inhibitory factor*) associated with reproductive traits. Two other genes identified on this same ROH segment, the *ZNRF3* (zinc and ring finger 3) and *KREMEN1* (kringle containing transmembrane protein 1), were associated with waist-hip ratio and fat distribution. The *METTL3* (methyltransferase like 3) gene found on ROH located on SSC7 is involved in the adipogenesis. The most shared ROH of the population (~47%) was located on chromosome SSC17 (between 17,836,349 and 20,730,659 bp), with length of 2.89 Mb, harboring 9 genes, of which the *TMX4* (thioredoxin-related transmembrane protein 4) and *PLCB1* (phospholipase C beta 1) were enriched in the superclusters negative regulation of hormone secretion and cerebral cortex development, respectively (Fig 5).

### Analysis of Population Structure

**Fig 6:**
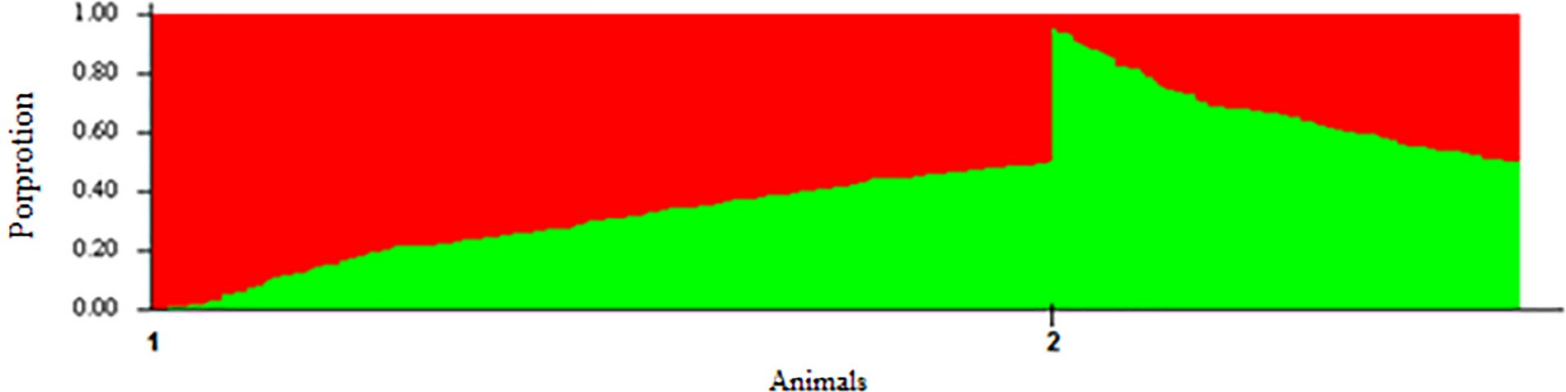
The genetic makeup of animals from the studied commercial maternal line using K = 2. The two colors represent the clusters formed according to the results: Red = Cluster 1 and Green = Cluster 2.

Two possible genetic group structures were revealed, represented by the delta-k value [14] (S1 Table). The largest portion (59%) of the population alleles were grouped in cluster 1 (Fig 6). The maximum and minimum values and mean allelic ratio for the animals sampled in cluster 1 were 0.996, 0.05 and 0.59 ± 0.23 respectively, while for cluster 2 the values were 0.95, 0.004 and 0.41 ± 0.22 (S2 Table), respectively. The results obtained from the likelihood procedure using the ADMIXTURE software were similar to those obtained in the Bayesian analysis, with the STRUCTURE software. Therefore, only the results of the STRUCTURE software are presented (S2 to S4 Figs). The average value of genomic Fst indicated that the degree of genetic divergence was 0.10 for the population studied.

**Fig 7:**
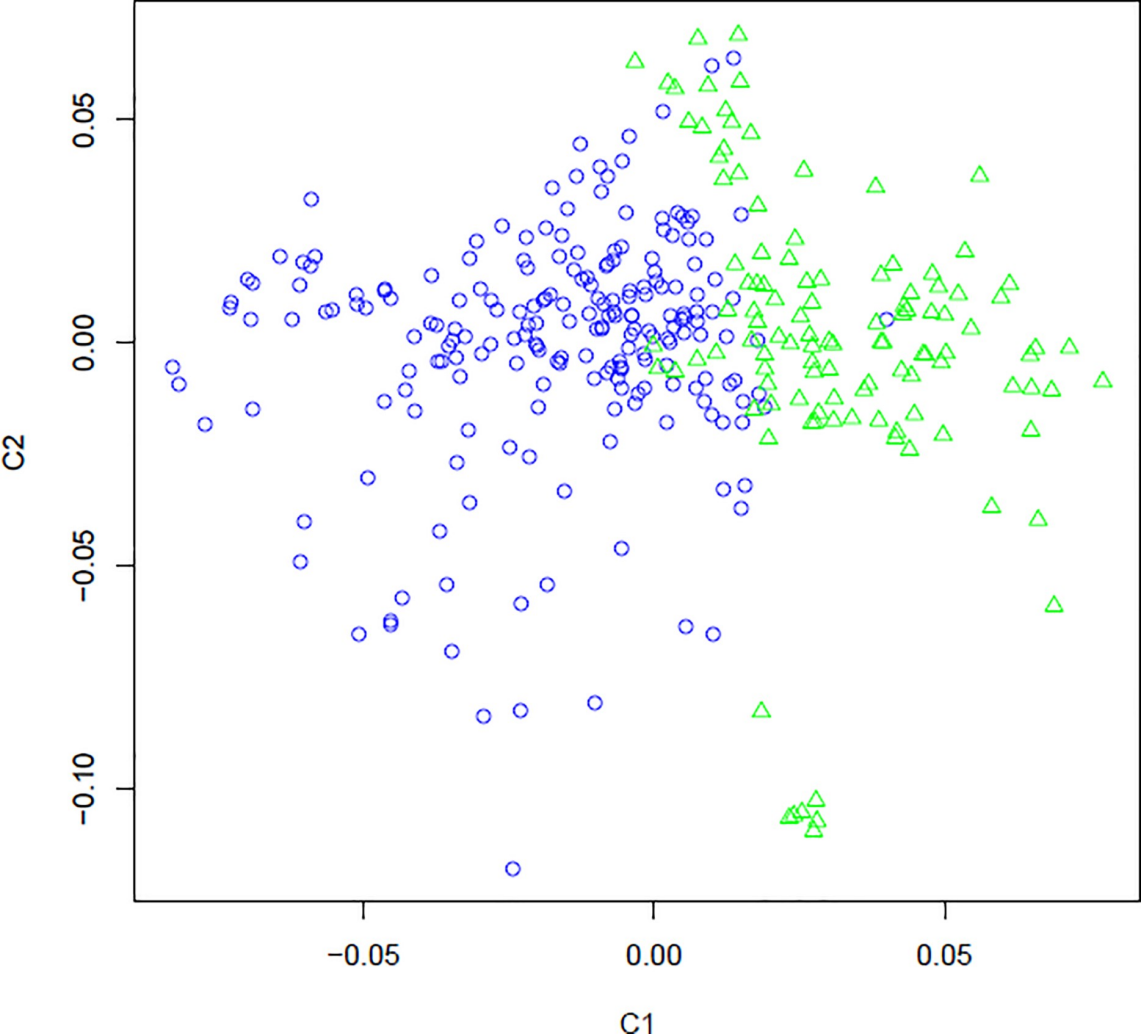
Population structure of a commercial maternal line revealed by the Principal Component Analysis for the SNP with r^2^ ≤ 0.20.

The results of the principal component analysis for all SNPs and for SNPs with LD less than 0.20 were similar. The population was divided into two clusters, based on the first principal component (Fig 7). The principal component can be interpreted as an index. Thereby for each individual was assigned an index value for each principal component, called an individual score of the principal component. From the visual analysis of Fig 6, an arbitrary value (0.02) for individual scores was obtained to categorize the clusters. Thus, individual scores higher or equal to 0.02 were placed in the green cluster, and animals with individual scores lower than 0.02 were placed in the blue cluster. Animals in the blue and green clusters have a higher proportion of eastern and western breeds, respectively.

## Discussion

A previous study with Landrace using the Illumina Porcine 60K BeadChip reported average r^2^ equal to 0.32 between markers with distance 0.5 Mb, excluding the SNPs for minor allelic frequency (MAF) and Hardy-Weinberg equilibrium criteria [18]. Badke et al. [19] used the software BEAGLE, excluding the same SNPs, reported average r^2^ equal to 0.19 for markers spaced 0.5 Mb in Landrace populations. These values are in agreement with the average r^2^ obtained in this study (0.29) for a Landrace-based pig line. Furthermore, Badke et al. [19] observed a higher average r^2^ for distances with less than 1 Mb. The LD decreasing pattern as the distances increase is expected because as the distance between markers on the same chromosome increase and the possibility of occurrence of recombination events (breaking of the linkage between the markers) increase as well.

The low correlation between inbreeding coefficient estimates based on ROH and pedigree could be the result of genetic recombination effect accounted for by the ROH estimates, once this effect cannot be considered to estimate the inbreeding coefficients from pedigree data. Moreover, estimates based on pedigree records neglect the effects of inbreeding accumulated over generations and it does not consider pedigree errors accumulated from past generations [5].

The correlation between F_ROH_ and F_PED_ found in this study (0.04) was lower than the 0.24 reported by Zanella et al. [9] in a Landrace population. Our results suggested that F_PED_ has been underestimated because the pedigree analysis considered three generations and possible animal identification errors may occurred.

A high number of long ROHs (> 5 Mb) may indicate that the population has not undergone recent crossings; otherwise, the crossing between different breeds would have caused these segments to breakdown. Herrero-Medrano et al. [8] reported mean ROH size between 50 and 100 Mb, considering long ROHs those greater than 100 Mb in Iberian breeds, while Bosse et al. [2] defined long ROHs those larger than 5 Mb. These authors studied wild and commercial pigs of the Asian and European continents and found a higher proportion of average ROH (0.1 to 5 Mb) in European commercial breeds. Similar results to this study have been reported for pure lines of Landrace and Large White, in which long ROHs with an approximate average of 252 Mb and 280 Mb, respectively, were observed [9].

The high prevalence of long ROHs can also indicate recent inbreeding of the population according to our results found for F_ROH_. Fisher [20] noted that the expected size of a DNA segment, which is identical by descent, follows the exponential distribution with mean equal to ½ g Morgans, where "g" is the number of past generations from the common ancestor. Therefore, long ROH (> 5 Mb) reflected inbreeding from a common ancestor, less than 10 past generations ago.

Inbreeding can lead to increased homozygous frequencies and risks of deleterious recessive genes to be co-expressed, causing inbreeding depression. The deleterious effects of inbreeding depression have been evaluated in several livestock species. Saura et al. [21] reported inbreeding depression in specific regions containing genes associated with litter size in Iberian pigs. Inbreeding depression was also observed in dairy cattle, where the increase of 1% of F_ROH_ resulted in a reduction of total milk yield to 205 days postpartum of 20 kg, increases in days open of 1.72 days and a decrease in some linear-type traits [22].

The high number of ROHs shared on SSC1 occurs because it is the largest chromosome of the pig genome, with a higher number of markers compared to shorter chromosomes (Table 1; Fig 3). The high number of ROH with low frequency identified at the end of the chromosomes may be due to recombination events (Fig 4). According to Tortereau et al. [23], recombination rates vary between and along chromosomes, and regions with high recombination rates tend to cluster close to the end of the chromosomes, regardless of the centromere position, as observed in this study.

The SSC17, which had the largest number of shared ROH of the population, has revealed the presence of QTLs affecting meat quality, carcass composition and signal peptide involved in stress reactions [24–25]. The ROHs identified in the present study present evidences of the selection history of this pig maternal line population. Twenty-one genes, including the *PLCB1* and *TMX4*, located on the most frequent ROH, were enriched in the superclusters negative regulation of hormone secretion, cerebral cortex development and locomotory behavior, which indicates possible selection for behavioral traits (Fig 5). The domestication pressure on *loci* that control fitness traits may favor the increase in the frequency of beneficial alleles creating homozygous regions in the genome of the studied population. Frantz et al. [26] reported that the genomes of domestic pigs have strong signatures of selection at *loci* that affect behavior and morphology, and still, that recurrent selection for traits related to domestication likely counteracted the homogenizing effect of gene flow from wild boars and created 'islands of domestication' in the genome.

Reproductive traits have been strongly selected in the last years to increase the production, especially in maternal lines, where the fertility and morphology of the sows are fundamental for a large litter size. Important genes were identified involving reproductive traits such as *LIF*, which plays a role on total number of piglets born in Landrace, Large White and Duroc breeds [27], and the genes *ZNRF3* and *KREMEN1*, which have been associated with waist-hip ratio and sexual dimorphism in the genetic basis of fat distribution [28]. Studies with pigs using other methodologies, have also identified significant portions of selection signatures that coincide with *loci* that control biological processes related to behavioral and reproductive traits, which may have been favored by the selection process [29–30].

The Fst value found in our study (0.10) was lower than the results reported by Herrero-Medrano et al. [8] in domestic and feral pigs in the Iberian Peninsula (0.22). The results indicated that approximately 10,000 SNPs would be sufficient to correct the effect of population stratification in potential genome-wide association studies in our population [16]. Moreover, Li et al. [31] using pair-wise Fst between Landrace and two Chinese indigenous pig breeds found Fst higher than we observed (Fst = 0.5480 for Erhualian and Fst = 0.5800 for Meishan), suggesting that Landrace had distant genetic relationship compared with the two Chinese breeds.

The results for K indicated the separation between the two genetic groups could be explained by the crossing between the Eastern and Western breeds used to form the studied population. The first cluster (population 1; Fig 6) grouped the animals with a higher proportion of Western breed alleles, while the second cluster (population 2; Fig 6) grouped the animals with a higher proportion of Eastern breed alleles. In the development of maternal lines of pigs, crossing between eastern and western breeds have been common. Maternal lines are developed to produce sows able to wean large numbers of piglets per litter per year. The western breeds Landrace and Large White and the eastern breeds, Meishan and Xia Jing, are commonly used as maternal lines due to high prolificacy and maternal ability [32].

As showed in Fig 6, the observed proportion of Eastern breed alleles was higher (~49%) than expected for this crossing (~25%), which can be attributed to genetic recombination events in the parental chromosomes that may occur during meiosis [33]. Moreover, the variation in the genetic makeup of animals regarding the allelic distribution of eastern and western breeds might be due to the selection of the sample, which sought to represent all the genetic variability of the studied population.

The results showed the importance of the SNP panels to provide knowledge on the population structure of the swine. The estimates of the inbreeding coefficient using genomic data indicated that the genetic diversity of the population studied should be frequently evaluated. Mating between less related individuals should be prioritized to ensure the efficiency of the breeding program applied to the population, to prevent inbreeding depression on traits of economic importance and to maintain genetic variation. Moreover, the study of the ROH regions of this population aids to understand of how the selective pressure and domestication can shape the genome during the animal domestication process, and it shows evidence that the ancestors of the studied population have undergone selective pressure for behavior and reproduction traits.

## Supporting information

S1 Table GO-terms associated with biological processes. (DOCX)

S2 Table Structure results—Allelic ratio per animal for each cluster, obtained with the Structure software. (DOCX)

S1 Fig Statistical delta-k based on the clustering method of Evanno et al. (TIF)

S2 Fig Genetic makeup of animals from the studied commercial maternal line for K = 3. Red = Cluster 1; Green = Cluster 2; Blue = Cluster 3.
(TIF)

S3 Fig Genetic makeup of animals from the studied commercial maternal line for K = 4. Red = Cluster 1; Green = Cluster 2; Blue = Cluster 3; Yellow = Cluster 4.
(TIF)

S4 Fig Genetic makeup of animals from the studied commercial maternal line for K = 5. Red = Cluster 1; Green = Cluster 2; Blue = Cluster 3; Yellow = Cluster 4; Pink = Cluster
(TIF)
