# Supplementary material for: Genomic structure of a crossbred Landrace pig population
Source: PLoS One. 2019 Feb 28;14(2):e0212266. doi: 10.1371/journal.pone.0212266 (PMC6394975; doi:10.1371/journal.pone.0212266)
Supplement: S1 Table — (DOCX) [file pone.0212266.s001.docx]

**S1 Table: GO-terms associated with biological processes**

|  | **Term** | **Genes** |
| --- | --- | --- |
| GO:0021987 | Cerebral cortex development | BTBD3, MKKS, PLCB1 |
| GO:0046888 | Negative regulation of hormone secretion | OSM, LIF |
| GO:0042503 | Tyrosine phosphorylation of Stat3 protein | OSM, LIF |
| GO:0009620 | Response to fungus | IL25, MALT1 |
| GO:0097094 | Craniofacial suture morphogenesis | RAB23, MMP16 |
| GO:0006457 | Protein folding | TMX4, BAG2, MKKS, CLPX |
| GO:0051603 | Proteolysis involved in cellular protein catabolic process | PSMB5, CLPX, PSMB11 |
| GO:0007626 | Locomotory behavior | PAK5, SNAP25, CALB1 |
| GO:0045835 | Negative regulation of meiotic nuclear division | OSM, LIF |
| GO:0042981 | Regulation of apoptotic process | BMP2, LOC100152318, MALT1, ACIN1 |
| GO:0007616 | Long-term memory | LRRN4, CALB1 |
| GO:0045746 | Negative regulation of Notch signaling pathway | BEND6, MMP14 |
| GO:0042517 | Positive regulation of tyrosine phosphorylation of Stat3 protein | OSM, LIF |
| GO:0040007 | Growth | BMP2, LOC100152318 |
| GO:0007612 | Learning | JPH4, PAK5 |
| GO:0001974 | Blood vessel remodeling | LIF, JAG1 |
| GO:0007613 | Memory | PAK5, PLCB1 |
