# Supplementary material for: Genomic structure of a crossbred Landrace pig population
Source: PLoS One. 2019 Feb 28;14(2):e0212266. doi: 10.1371/journal.pone.0212266 (PMC6394975; doi:10.1371/journal.pone.0212266)
Supplement: S2 Table — (DOCX) [file pone.0212266.s002.docx]

**S2 Table: Structure results - Allelic ratio per animal for each cluster, obtained with the Structure software.**

| Animal | Cluster 1 | Cluster 2 |  |
| --- | --- | --- | --- |
| AN1 | 0.654 | 0.346 | |
| AN2 | 0.464 | 0.536 | |
| AN3 | 0.623 | 0.377 | |
| AN4 | 0.501 | 0.499 | |
| AN5 | 0.523 | 0.477 | |
| AN6 | 0.473 | 0.527 | |
| AN7 | 0.845 | 0.155 | |
| AN8 | 0.692 | 0.308 | |
| AN9 | 0.681 | 0.319 | |
| AN10 | 0.660 | 0.340 | |
| AN11 | 0.819 | 0.181 | |
| AN12 | 0.498 | 0.502 | |
| AN13 | 0.990 | 0.010 | |
| AN14 | 0.541 | 0.459 | |
| AN15 | 0.918 | 0.082 | |
| AN16 | 0.414 | 0.586 | |
| AN17 | 0.876 | 0.124 | |
| AN18 | 0.903 | 0.097 | |
| AN19 | 0.688 | 0.312 | |
| AN20 | 0.770 | 0.230 | |
| AN21 | 0.809 | 0.191 | |
| AN22 | 0.628 | 0.372 | |
| AN23 | 0.608 | 0.392 | |
| AN24 | 0.632 | 0.368 | |
| AN25 | 0.665 | 0.335 | |
| AN26 | 0.610 | 0.390 | |
| AN27 | 0.516 | 0.484 | |
| AN28 | 0.570 | 0.430 | |
| AN29 | 0.570 | 0.430 | |
| AN30 | 0.783 | 0.217 | |
| AN31 | 0.213 | 0.787 | |
| AN32 | 0.488 | 0.512 | |
| AN33 | 0.666 | 0.334 | |
| AN34 | 0.641 | 0.359 | |
| AN35 | 0.308 | 0.692 | |
| AN36 | 0.174 | 0.826 | |
| AN37 | 0.806 | 0.194 | |
| AN38 | 0.316 | 0.684 | |
| AN39 | 0.819 | 0.181 | |
| AN40 | 0.533 | 0.467 | |
| AN41 | 0.833 | 0.167 | |
| AN42 | 0.326 | 0.674 | |
| AN43 | 0.315 | 0.685 | |
| AN44 | 0.590 | 0.410 | |
| AN45 | 0.657 | 0.343 | |
| AN46 | 0.461 | 0.539 | |
| AN47 | 0.401 | 0.599 | |
| AN48 | 0.476 | 0.524 | |
| AN49 | 0.395 | 0.605 | |
| AN50 | 0.536 | 0.464 | |
| AN51 | 0.473 | 0.527 | |
| AN52 | 0.506 | 0.494 | |
| AN53 | 0.617 | 0.383 | |
| AN54 | 0.628 | 0.372 | |
| AN55 | 0.529 | 0.471 | |
| AN56 | 0.518 | 0.482 | |
| AN57 | 0.689 | 0.311 | |
| AN58 | 0.994 | 0.006 | |
| AN59 | 0.670 | 0.330 | |
| AN60 | 0.439 | 0.561 | |
| AN61 | 0.993 | 0.007 | |
| AN62 | 0.552 | 0.448 | |
| AN63 | 0.364 | 0.636 | |
| AN64 | 0.795 | 0.205 | |
| AN65 | 0.515 | 0.485 | |
| AN66 | 0.208 | 0.792 | |
| AN67 | 0.361 | 0.639 | |
| AN68 | 0.656 | 0.344 | |
| AN69 | 0.327 | 0.673 | |
| AN70 | 0.975 | 0.025 | |
| AN71 | 0.592 | 0.408 | |
| AN72 | 0.593 | 0.407 | |
| AN73 | 0.886 | 0.114 | |
| AN74 | 0.551 | 0.449 | |
| AN75 | 0.440 | 0.560 | |
| AN76 | 0.148 | 0.852 | |
| AN77 | 0.350 | 0.650 | |
| AN78 | 0.386 | 0.614 | |
| AN79 | 0.996 | 0.004 | |
| AN80 | 0.992 | 0.008 | |
| AN81 | 0.296 | 0.704 | |
| AN82 | 0.491 | 0.509 | |
| AN83 | 0.487 | 0.513 | |
| AN84 | 0.308 | 0.692 | |
| AN85 | 0.125 | 0.875 | |
| AN86 | 0.647 | 0.353 | |
| AN87 | 0.445 | 0.555 | |
| AN88 | 0.120 | 0.880 | |
| AN89 | 0.938 | 0.062 | |
| AN90 | 0.553 | 0.447 | |
| AN91 | 0.982 | 0.018 | |
| AN92 | 0.520 | 0.480 | |
| AN93 | 0.943 | 0.057 | |
| AN94 | 0.593 | 0.407 | |
| AN95 | 0.882 | 0.118 | |
| AN96 | 0.613 | 0.387 | |
| AN97 | 0.687 | 0.313 | |
| AN98 | 0.731 | 0.269 | |
| AN99 | 0.423 | 0.577 | |
| AN100 | 0.704 | 0.296 | |
| AN101 | 0.548 | 0.452 | |
| AN102 | 0.582 | 0.418 | |
| AN103 | 0.596 | 0.404 | |
| AN104 | 0.185 | 0.815 | |
| AN105 | 0.269 | 0.731 | |
| AN106 | 0.541 | 0.459 | |
| AN107 | 0.114 | 0.886 | |
| AN108 | 0.767 | 0.233 | |
| AN109 | 0.723 | 0.277 | |
| AN110 | 0.540 | 0.460 | |
| AN111 | 0.259 | 0.741 | |
| AN112 | 0.393 | 0.607 | |
| AN113 | 0.336 | 0.664 | |
| AN114 | 0.754 | 0.246 | |
| AN115 | 0.367 | 0.633 | |
| AN116 | 0.376 | 0.624 | |
| AN117 | 0.612 | 0.388 | |
| AN118 | 0.660 | 0.340 | |
| AN119 | 0.403 | 0.597 | |
| AN120 | 0.759 | 0.241 | |
| AN121 | 0.589 | 0.411 | |
| AN122 | 0.531 | 0.469 | |
| AN123 | 0.507 | 0.493 | |
| AN124 | 0.316 | 0.684 | |
| AN125 | 0.060 | 0.940 | |
| AN126 | 0.779 | 0.221 | |
| AN127 | 0.466 | 0.534 | |
| AN128 | 0.534 | 0.466 | |
| AN129 | 0.138 | 0.862 | |
| AN130 | 0.898 | 0.102 | |
| AN131 | 0.778 | 0.222 | |
| AN132 | 0.844 | 0.156 | |
| AN133 | 0.463 | 0.537 | |
| AN134 | 0.848 | 0.152 | |
| AN135 | 0.966 | 0.034 | |
| AN136 | 0.525 | 0.475 | |
| AN137 | 0.990 | 0.010 | |
| AN138 | 0.853 | 0.147 | |
| AN139 | 0.651 | 0.349 | |
| AN140 | 0.563 | 0.437 | |
| AN141 | 0.497 | 0.503 | |
| AN142 | 0.888 | 0.112 | |
| AN143 | 0.650 | 0.350 | |
| AN144 | 0.675 | 0.325 | |
| AN145 | 0.715 | 0.285 | |
| AN146 | 0.183 | 0.817 | |
| AN147 | 0.940 | 0.060 | |
| AN148 | 0.830 | 0.170 | |
| AN149 | 0.325 | 0.675 | |
| AN150 | 0.752 | 0.248 | |
| AN151 | 0.822 | 0.178 | |
| AN152 | 0.574 | 0.426 | |
| AN153 | 0.880 | 0.120 | |
| AN154 | 0.924 | 0.076 | |
| AN155 | 0.068 | 0.932 | |
| AN156 | 0.924 | 0.076 | |
| AN157 | 0.859 | 0.141 | |
| AN158 | 0.372 | 0.628 | |
| AN159 | 0.743 | 0.257 | |
| AN160 | 0.745 | 0.255 | |
| AN161 | 0.511 | 0.489 | |
| AN163 | 0.405 | 0.595 | |
| AN164 | 0.286 | 0.714 | |
| AN165 | 0.364 | 0.636 | |
| AN166 | 0.728 | 0.272 | |
| AN167 | 0.100 | 0.900 | |
| AN168 | 0.943 | 0.057 | |
| AN169 | 0.699 | 0.301 | |
| AN170 | 0.742 | 0.258 | |
| AN171 | 0.399 | 0.601 | |
| AN172 | 0.734 | 0.266 | |
| AN173 | 0.449 | 0.551 | |
| AN174 | 0.526 | 0.474 | |
| AN175 | 0.537 | 0.463 | |
| AN176 | 0.311 | 0.689 | |
| AN177 | 0.625 | 0.375 | |
| AN178 | 0.736 | 0.264 | |
| AN179 | 0.596 | 0.404 | |
| AN180 | 0.623 | 0.377 | |
| AN181 | 0.783 | 0.217 | |
| AN182 | 0.643 | 0.357 | |
| AN183 | 0.490 | 0.510 | |
| AN184 | 0.512 | 0.488 | |
| AN185 | 0.726 | 0.274 | |
| AN186 | 0.803 | 0.197 | |
| AN187 | 0.447 | 0.553 | |
| AN188 | 0.684 | 0.316 | |
| AN189 | 0.785 | 0.215 | |
| AN190 | 0.422 | 0.578 | |
| AN191 | 0.262 | 0.738 | |
| AN192 | 0.695 | 0.305 | |
| AN193 | 0.420 | 0.580 | |
| AN194 | 0.509 | 0.491 | |
| AN195 | 0.983 | 0.017 | |
| AN196 | 0.761 | 0.239 | |
| AN197 | 0.320 | 0.680 | |
| AN198 | 0.920 | 0.080 | |
| AN199 | 0.741 | 0.259 | |
| AN200 | 0.690 | 0.310 | |
| AN201 | 0.521 | 0.479 | |
| AN202 | 0.827 | 0.173 | |
| AN203 | 0.242 | 0.758 | |
| AN204 | 0.873 | 0.127 | |
| AN205 | 0.789 | 0.211 | |
| AN206 | 0.779 | 0.221 | |
| AN207 | 0.557 | 0.443 | |
| AN208 | 0.058 | 0.942 | |
| AN209 | 0.655 | 0.345 | |
| AN210 | 0.551 | 0.449 | |
| AN211 | 0.224 | 0.776 | |
| AN212 | 0.742 | 0.258 | |
| AN213 | 0.802 | 0.198 | |
| AN214 | 0.608 | 0.392 | |
| AN215 | 0.760 | 0.240 | |
| AN216 | 0.984 | 0.016 | |
| AN217 | 0.968 | 0.032 | |
| AN218 | 0.063 | 0.937 | |
| AN219 | 0.102 | 0.898 | |
| AN220 | 0.572 | 0.428 | |
| AN221 | 0.611 | 0.389 | |
| AN222 | 0.557 | 0.443 | |
| AN223 | 0.643 | 0.357 | |
| AN224 | 0.270 | 0.730 | |
| AN225 | 0.625 | 0.375 | |
| AN226 | 0.548 | 0.452 | |
| AN227 | 0.269 | 0.731 | |
| AN228 | 0.604 | 0.396 | |
| AN229 | 0.589 | 0.411 | |
| AN230 | 0.844 | 0.156 | |
| AN231 | 0.710 | 0.290 | |
| AN232 | 0.464 | 0.536 | |
| AN233 | 0.543 | 0.457 | |
| AN234 | 0.772 | 0.228 | |
| AN235 | 0.332 | 0.668 | |
| AN236 | 0.942 | 0.058 | |
| AN237 | 0.851 | 0.149 | |
| AN238 | 0.785 | 0.215 | |
| AN239 | 0.196 | 0.804 | |
| AN240 | 0.349 | 0.651 | |
| AN241 | 0.510 | 0.490 | |
| AN242 | 0.580 | 0.420 | |
| AN243 | 0.992 | 0.008 | |
| AN244 | 0.992 | 0.008 | |
| AN245 | 0.652 | 0.348 | |
| AN246 | 0.653 | 0.347 | |
| AN247 | 0.874 | 0.126 | |
| AN248 | 0.498 | 0.502 | |
| AN249 | 0.312 | 0.688 | |
| AN250 | 0.332 | 0.668 | |
| AN251 | 0.983 | 0.017 | |
| AN252 | 0.993 | 0.007 | |
| AN253 | 0.885 | 0.115 | |
| AN254 | 0.527 | 0.473 | |
| AN255 | 0.331 | 0.669 | |
| AN256 | 0.785 | 0.215 | |
| AN257 | 0.416 | 0.584 | |
| AN258 | 0.780 | 0.220 | |
| AN259 | 0.557 | 0.443 | |
| AN260 | 0.723 | 0.277 | |
| AN261 | 0.331 | 0.669 | |
| AN262 | 0.251 | 0.749 | |
| AN263 | 0.680 | 0.320 | |
| AN264 | 0.132 | 0.868 | |
| AN265 | 0.762 | 0.238 | |
| AN266 | 0.407 | 0.593 | |
| AN267 | 0.733 | 0.267 | |
| AN268 | 0.176 | 0.824 | |
| AN269 | 0.679 | 0.321 | |
| AN270 | 0.446 | 0.554 | |
| AN271 | 0.116 | 0.884 | |
| AN272 | 0.050 | 0.950 | |
| AN273 | 0.633 | 0.367 | |
| AN274 | 0.616 | 0.384 | |
| AN275 | 0.585 | 0.415 | |
| AN276 | 0.774 | 0.226 | |
| AN277 | 0.762 | 0.238 | |
| AN278 | 0.782 | 0.218 | |
| AN279 | 0.797 | 0.203 | |
| AN280 | 0.783 | 0.217 | |
| AN281 | 0.446 | 0.554 | |
| AN282 | 0.299 | 0.701 | |
| AN283 | 0.471 | 0.529 | |
| AN284 | 0.451 | 0.549 | |
| AN285 | 0.338 | 0.662 | |
| AN286 | 0.511 | 0.489 | |
| AN287 | 0.553 | 0.447 | |
| AN288 | 0.866 | 0.134 | |
| AN289 | 0.176 | 0.824 | |
| AN290 | 0.775 | 0.225 | |
| AN291 | 0.696 | 0.304 | |
| AN292 | 0.714 | 0.286 | |
| AN293 | 0.748 | 0.252 | |
| AN294 | 0.727 | 0.273 | |
| AN295 | 0.606 | 0.394 | |
| AN296 | 0.637 | 0.363 | |
| AN297 | 0.727 | 0.273 | |
| AN298 | 0.582 | 0.418 | |
| AN299 | 0.754 | 0.246 | |
| AN300 | 0.740 | 0.260 | |
| AN301 | 0.257 | 0.743 | |
| AN302 | 0.495 | 0.505 | |
| AN303 | 0.500 | 0.500 | |
| AN304 | 0.383 | 0.617 | |
| AN305 | 0.459 | 0.541 | |
| AN306 | 0.553 | 0.447 | |
| AN307 | 0.491 | 0.509 | |
| AN308 | 0.453 | 0.547 | |
| AN309 | 0.091 | 0.909 | |
| AN310 | 0.945 | 0.055 | |
| AN311 | 0.249 | 0.751 | |
| AN312 | 0.389 | 0.611 | |
| AN313 | 0.969 | 0.031 | |
| AN314 | 0.315 | 0.685 | |
| AN315 | 0.345 | 0.655 | |
| AN316 | 0.183 | 0.817 | |
| AN317 | 0.403 | 0.597 | |
| AN318 | 0.698 | 0.302 | |
| AN319 | 0.463 | 0.537 | |
| AN320 | 0.319 | 0.681 | |
| AN321 | 0.509 | 0.491 | |
| AN322 | 0.555 | 0.445 | |
| AN323 | 0.509 | 0.491 | |
| AN324 | 0.380 | 0.620 | |
| AN325 | 0.530 | 0.470 | |
| AN326 | 0.753 | 0.247 | |
