# Supplementary figures and images for: Genomic structure of a crossbred Landrace pig population
Source: PLoS One. 2019 Feb 28;14(2):e0212266. doi: 10.1371/journal.pone.0212266 (PMC6394975; doi:10.1371/journal.pone.0212266)

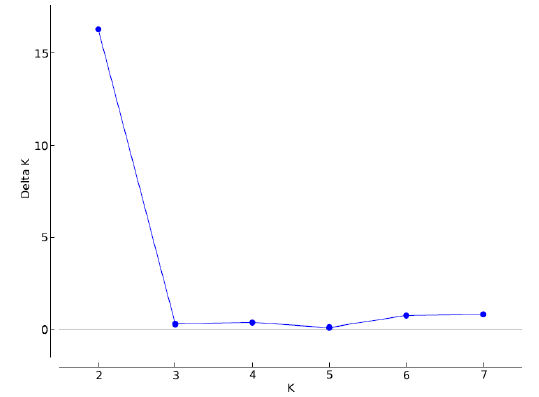

Supplement: S1 Fig — (TIF) [file pone.0212266.s003.tif]

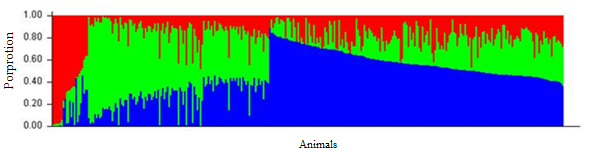

Supplement: S2 Fig — Red = Cluster 1; Green = Cluster 2; Blue = Cluster 3. (TIF) [file pone.0212266.s004.tif]

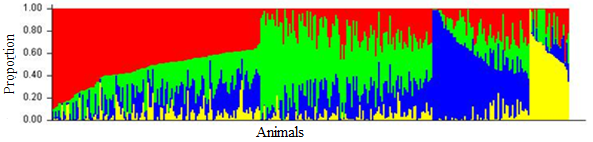

Supplement: S3 Fig — Red = Cluster 1; Green = Cluster 2; Blue = Cluster 3; Yellow = Cluster 4. (TIF) [file pone.0212266.s005.tif]

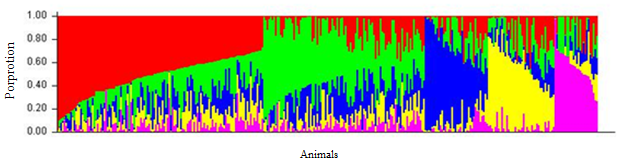

Supplement: S4 Fig — Red = Cluster 1; Green = Cluster 2; Blue = Cluster 3; Yellow = Cluster 4; Pink = Cluster (TIF) [file pone.0212266.s006.tif]
